# A novel leptin antagonist peptide inhibits breast cancer growth *in vitro* and *in vivo*

**DOI:** 10.1111/jcmm.12517

**Published:** 2015-02-27

**Authors:** Stefania Catalano, Antonella Leggio, Ines Barone, Rosaria De Marco, Luca Gelsomino, Antonella Campana, Rocco Malivindi, Salvatore Panza, Cinzia Giordano, Alessia Liguori, Daniela Bonofiglio, Angelo Liguori, Sebastiano Andò

**Affiliations:** a Department of Pharmacy, Health and Nutritional Sciences, University of CalabriaArcavacata di Rende, CS, Italy; b Breast Center, Baylor College of MedicineHouston, TX, USA; c Centro Sanitario, University of CalabriaArcavacata di Rende CS, Italy; d Department of Medical Oncology, University of MessinaMessina, Italy

**Keywords:** leptin, leptin receptor, leptin receptor modulators, breast cancer, peptides

## Abstract

The role of the obesity cytokine leptin in breast cancer progression has raised interest in interfering with leptin's actions as a valuable therapeutic strategy. Leptin interacts with its receptor through three different binding sites: I–III. Site I is crucial for the formation of an active leptin–leptin receptor complex and in its subsequent activation. Amino acids 39-42 (Leu-Asp-Phe-Ile- LDFI) were shown to contribute to leptin binding site I and their mutations in alanine resulted in muteins acting as typical antagonists. We synthesized a small peptide based on the wild-type sequence of leptin binding site I (LDFI) and evaluated its efficacy in antagonizing leptin actions in breast cancer using *in vitro* and *in vivo* experimental models. The peptide LDFI abolished the leptin-induced anchorage-dependent and -independent growth as well as the migration of ERα-positive (MCF-7) and -negative (SKBR3) breast cancer cells. These results were well correlated with a reduction in the phosphorylation levels of leptin downstream effectors, as JAK2/STAT3/AKT/MAPK. Importantly, the peptide LDFI reversed the leptin-mediated up-regulation of its gene expression, as an additional mechanism able to enhance the peptide antagonistic activity. The described effects were specific for leptin signalling, since the developed peptide was not able to antagonize the other growth factors' actions on signalling activation, proliferation and migration. Finally, we showed that the LDFI pegylated peptide markedly reduced breast tumour growth in xenograft models. The unmodified peptide LDFI acting as a full leptin antagonist could become an attractive option for breast cancer treatment, especially in obese women.

## Introduction

Carcinoma of the breast is the most common cancer among women in industrialized countries, and results in substantial morbidity and mortality. Breast carcinogenesis is thought to involve genetic predisposition, but modifiable factors such as overweight (body mass index, BMI 25–30 kg/m^2^) or obesity (BMI >30 kg/m^2^) conditions have also been reported to play an important role 1,2. Indeed, a number of epidemiological studies have suggested that obesity and high adipose tissue mass are associated with an increased risk of breast cancer development, as well as with an aggressive tumour phenotype and a poor survival 3–5. Among obesity-related factors that are known to impact breast cancer development and progression, the adipose-derived adipokine leptin, whose synthesis and plasma levels increase proportionally to total adipose tissue mass 6,7 has been extensively examined in this regard.

Leptin, a 16 kD polypeptide hormone encoded by the obese (*Ob*) gene, is a pleiotropic molecule that regulates food intake, hematopoiesis, inflammation, cell differentiation and proliferation, but it is also required for mammary gland development and tumourigenesis 8. Both leptin and its receptor (ObR) are overexpressed in breast cancer, especially in higher grade tumours and are associated with distant metastasis 9,10. Genetically obese leptin-deficient Lep^ob/ob^ and leptin receptor-deficient Lepr^db/db^ mice do not develop mammary tumours which provide evidence that leptin and its receptor are involved in breast tumourigenesis 11,12. In line with these observations, a growing body of evidence have shown that leptin is able to induce a variety of responses, such as mitogenesis, survival, transformation, migration and invasion in breast cancer cells 13–20 through the activation of several signalling pathways, such as those involving Janus kinase 2–signal transducer and activator of transcription 3 (JAK2-STAT3), mitogen-activated protein kinase (MAPK), and phosphatidylinositol 3-kinase-protein kinase B (PI3K-AKT) 21. In addition to its direct action, we and other authors have demonstrated that leptin can exert its tumourigenic activities also interacting with different signalling molecules. Indeed, leptin signalling in human breast cancer cells enhances aromatase gene expression promoting *in situ* oestrogen production 22 and directly transactivates oestrogen receptor alpha (ERα) 17,23. It has also been reported an interplay between leptin signalling and the transmembrane tyrosine kinase receptor HER2, a member of epidermal growth factor receptor (EGFR) family 24–26. Saxena *et al*. have demonstrated the existence of a bidirectional crosstalk between leptin and insulin-like growth factor I (IGF-I) signalling, mediated by synergistic transactivation of EGFR, which influences breast cancer cell invasion and migration 27. Of note, we have demonstrated that leptin acting as a mediator of tumour /stroma interaction within tumour microenvironment may promote mammary carcinogenesis 8,17.

Several potential therapeutic approaches able to inhibit leptin activity, especially in obese cancer patients, have been proposed. Antagonists to the leptin receptor are being developed both as mutants of the full protein and peptide fragments representing single receptor-binding site 20,28–30. In this regard, it has been previously demonstrated that inhibition of leptin signalling by a pegylated leptin peptide receptor antagonist (PEG-LPrA2), a short peptide corresponding to amino acids 70-95 of human leptin, resulted in decreased growth of mammary tumours derived from mice and humans 31–33. Similar data were reported by another group using a different leptin antagonist, a 9 residue peptidomimetic named as Allo-aca. This peptide inhibits leptin-mediated proliferation and signalling *in vitro* and exhibits anti-neoplastic activities *in vivo* 34,35.

In this study, we have generated a novel peptide, LDFI, corresponding to amino acid residues 39-42 from one of the putative sites of interaction of leptin with its receptor. Our results have shown that LDFI inhibits leptin-induced proliferation and motility as well as leptin signalling activation in both ERα-positive and ERα-negative human breast cancer cells. Furthermore, the peptide markedly reduces breast tumour growth in xenograft models.

## Materials and methods

### Reagents and antibodies

DMEM, McCoy's 5A Medium, L-Glutamine, penicillin, streptomycin, bovine serum albumin (BSA), phosphate-buffered saline, TRIzol, 100 bp DNA ladder were purchased from Invitrogen (Carlsbad, CA, USA). TaqDNA polymerase was provided by Promega (Madison, WI, USA). The RETROscript kit and DNase I were purchased from Ambion (Austin, TX, USA). Aprotinin, leupeptin, phenylmethylsulfonyl fluoride, sodium orthovanadate, formaldehyde, NP-40, MTT, dimethyl sulphoxide, proteinase K, leptin and epidermal growth factor (EGF) by Sigma-Aldrich (Milan, Italy).

Cyclin D1, GAPDH, total Akt and phosphorylated pAkt (Ser^437^), Ki-67 antibodies were from Santa Cruz Biotechnology (Santa Cruz, CA, USA). Total ERK1,2/MAPK, JAK2, STAT3 and phosphorylated p42/44 ERK1,2/MAPK (Thr^202^/Tyr^204^), JAK2 (Tyr^1007/1008^), STAT3 (Tyr^705^) were from Cell Signaling Technology (Beverly, MA, USA).

### Peptide synthesis

The peptides were synthesized by solid phase methods using reagent systems and methodologies of standard Fmoc-chemistry 36,37. Fmoc-L-Asp-(OtBu)-OH, Wang resin, diisopropylethylamine (DIPEA), trifluoroacetic acid (TFA) and triisopropylsilane (TIS) were purchased from Sigma-Aldrich. All other Fmoc-protected amino acids (Fmoc-L-AA-OH) were prepared from the corresponding α-amino acids and 9-fluorenylmethyloxycarbonyl chloride 38. O-Benzotriazole-*N*,*N*,*N*′,*N*′-tetramethyl-uronium-hexafluoro-phosphate (HBTU) was purchased from Matrix Innovation.

For the synthesis of polyethylene glycol (PEG)-attached peptide was used TentaGel® PAP Resin (Rapp Polymere GmbH, Tuebingen, Germany), a polystyrene/DVB matrix with amine terminated polyethylene oxide attached *via* a cleavable linkage with TFA. Dichloromethane (DCM), *N*,*N*-dimethylformamide (DMF), *N*-methylpyrrolidone (NMP), diethyl ether, formic acid (FA), HPLC grade water and acetonitrile were obtained by VWR.

The peptides were characterized by NMR spectroscopy and LC/MS analysis. Electrospray ionization source-quadrupole time of flight (ESI-QTOF) mass spectra and ^1^H NMR and ^13^C NMR spectra identified correct and pure samples (more details in Data S1).

### ESI-QTOF mass spectra were recorded on a Agilent 6540 Q-TOF mass spectrometer

^1^H NMR and ^13^C NMR spectra were recorded on a Bruker Avance 300 spectrometer using DMSO-d_6_ as solvent. Chemical shifts (δ) are reported in units of parts per million (ppm) and all coupling constants (J) are reported in hertz (Hz). LC-MS analysis was carried out using a UHPLC instrument coupled to a QTOF mass spectrometer fitted with a ESI operating in positive ion mode. Chromatographic separation was achieved using a C18 RP analytical column (Eclipse Plus C18, 50 × 2.1 mm, 1.8 μm) at 50°C with a elution gradient from 5% to 50% of B over 15 min., A being H_2_O (0.1% FA) and B CH_3_CN (0.1% FA). Flow rate was 0.5 ml/min.

Standard microwave synthesis protocol: the peptide chain assembly was made on a CEM-Liberty microwave-assisted automated synthesizer. The resin was swollen in DMF for 30 min. before use. Coupling reactions were performed in DMF with fivefold excess of Fmoc-AA-OH in the presence of HBTU (5.0 equivalents) and DIPEA (10 equivalents) for 5 min. at 75°C. The Fmoc protecting group was removed by treatment of the resin with a 20% solution of piperidine in DMF (v/v) (7 ml). Deprotection was performed in two stages with an initial deprotection of 30 sec. followed by 3 min. at 75°C. Between each step the resin was washed thoroughly with DMF. The completed peptide was washed with DMF and DCM. The peptide was cleaved from the resin using a mixture of TFA, water, and TIS (9.5/0.25/0.25 by volume) for 30 min. at 38 °C. Following cleavage the peptide was precipitated and washed with diethyl ether.

#### LDFI peptide

**ESI-QTOF-MS:** 507.2814 (M+H)^+^, 529.2636 (M+Na)^+^, 545.2367 (M+K)^+^.

**ESI-QTOF-MS:** calcd for C_25_H_39_N_4_O_7_^+^ 507.2813, found 507.2814.

#### Scramble peptide LLLA

**ESI-QTOF-MS:** 429.3078 (M+H)^+^, 451.2899 (M+Na)^+^, 467.2576 (M+K)^+^.

**ESI-QTOF-MS:** calcd for C_21_H_41_N_4_O_5_^+^ 429.3071, found 429.3078.

#### LDFI-PEG peptide

LDFI-PEG peptide was characterized by LC-ESI-QTOF-MS analysis which shows that the peptide LDFI is linked to PEG. The chromatogram shows a peak eluting at 3.21 min. that comprises a mass of 626.3514 with z = 2. The MS/MS spectrum of the peak at 3.21 min. (m/z 626.3514, z = 2) confirmed the structure of an adduct between LDFI and PEG.

**ESI-QTOF MS/MS: (626.3514; z = 2):** 376.1824 (b_3_- H_2_O), 229.1173 (b_2_), 86.0963 (L,I).

### Cell cultures

The human breast cancer cell lines MCF-7 and SKBR3 were acquired from American Type Culture Collection (Manassas, VA, USA) where they were authenticated, stored according to supplier's instructions, and used within a month after frozen aliquots resuscitations. MCF-7 cells were cultured in DMEM medium supplemented with 10% foetal bovine serum (FBS; Invitrogen), 2 mmol/l L-glutamine, and 50 units/ml penicillin/streptomycin. SKBR3 cells were cultured in McCoy's 5A Medium modified containing 10% FBS, 1% L-glutamine, 1% Eagle's nonessential amino acids, and 1 mg/ml penicillin-streptomycin. Before each experiment, cells were grown in phenol red-free medium, containing 5% charcoal-stripped FBS for 2 days and treated as described.

### Cell proliferation assays

#### MTT anchorage-dependent growth assays

Cell viability was determined using the 3-(4,5-dimethylthiazol-2-yl)-2,5-diphenyltetrazolium (MTT) assay as described previously 39. Results are representative of at least three independent experiments and expressed as the absorbance readings at 570 nm.

#### Soft agar anchorage-independent growth assays

Cells (10^4^/well) were plated in 4 ml of 0.35% agarose with 5% charcoal stripped-FBS in phenol red-free media, with a 0.7% agarose base in six well plates. Two days after plating, media containing vehicle or treatments, as indicated, were added to the top layer and replaced every 2 days. After 14 days, colonies were counted as described 40. Data shown are the mean colony numbers of three independent experiments each performed in triplicate.

#### Wound-healing migration assays

Motility was assessed as described previously 17. Briefly, cell monolayers were scraped and cells were treated as indicated. Closure of the wound was monitored over 24 hrs, cells were then fixed and stained with Comassie Brillant Blue. Pictures were taken at 10× magnifications using phase-contrast microscopy and are representative of three independent experiments.

#### Transmigration assays

Cells treated as indicated were placed in the upper compartments of Boyden chamber (8-μm membranes; Corning Costar, NY, USA). Bottom well contained regular full media. After 24 hrs, migrated cells were fixed and stained with Coomassie brilliant blue. Migration was quantified by viewing 5 separate fields per membrane at 20× magnification and expressed as the mean number of migrated cells. Data represent 3 independent experiments assayed in triplicate.

#### Reverse transcription-PCR and real-time RT-PCR assays

The gene expression of Ob (leptin) and 36B4 was evaluated by reverse transcription PCR (RT-PCR) method as previously described 41, using the following primers: 5′-GAGACCTCCTCCATGTGCTG-3′ (Ob forward) and 5′-TGAGCTCAGATATCGGGCTGAAC-3′ (Ob reverse), 5′-CTCAACATCTCCCCCTTCTC-3′ (36B4 forward) and 5′-CAAATCCCATATCCTCGT-3′ (36B4 reverse). Negative control contained water instead of first strand cDNA was used.

The gene expression of the long and short leptin receptor isoforms (ObRL/ObRsh), VEGF receptor (VEGFR) was assessed by real-time RT-PCR, using SYBR Green Universal PCR Master Mix (Bio-Rad, Hercules, CA, USA). Each sample was normalized on GAPDH mRNA content. Primers used for the amplification were: forward 5′-ATTGTGCCAGTAATTATT TCCTCTTCC-3′ and reverse 5′-CCACCATATGTTAAC TCTCAGAAGTTCAA-3′ (ObRl), forward 5′-GATAGA GGCCCAGGCATTTTTTA-3′ and reverse 5′-ACACCACTCTCTCTCTTTTTGATTGA-3′ (ObRs), forward 5′-CTTCGAAGCATCAGCATAAGAAACT-3′ and reverse 5′-TGGTCAGCC CACTGGAT-3′ (VEGFR), forward 5′-CCCACTCCTCCACCTTTGAC-3′ and reverse 5′-TGTTGCTGTAGCCAA ATTCGT T-3′ (GADPDH). The relative gene expression levels were normalized to a calibrator that was chosen to be the basal, vehicle-treated sample. Final results were expressed as n-fold differences in gene expression relative to GAPDH rRNA and calibrator, calculated using the ΔΔCt method as follows: n-fold = 2^−(ΔCtsample−ΔCtcalibrator)^, where ΔCt values of the sample and calibrator were determined by subtracting the average Ct value of the GAPDH rRNA reference gene from the average Ct value of the different genes analyzed.

### Immunoblotting analysis

Whole-cell lysates were prepared as previously described 42. Protein extracts from tumour tissues were prepared as previously described 43. Equal amounts of total protein were resolved on 11% SDS-PAGE as indicated 44. Blots shown are representative of at least three independent experiments.

### Tumour xenografts

*In vivo* studies were conducted in 45-day-old female nude mice (nu/nu Swiss). Mice were inoculated with exponentially growing SKBR3 cells (5.0 × 10^6^ per mouse) in 0.1 ml of matrigel (BD Biosciences, Bedford, MA, USA) into the intrascapular region. Once tumours reached an approximate volume of 100 mm^3^ 5 mice/group were randomly allocated into three groups. The mice were then treated with LDFI-PEG (1 and 10 mg/kg/day) diluted in saline 0.3% BSA or saline 0.3% BSA only (control) by i.p. injection. The treatment was done for 5 days a week until the 4th week. All animals were maintained and handled in accordance with the recommendation of the Guidelines for the Care and Use of Laboratory Animals and were approved by the Animal Care Committee of University of Calabria. Tumour development was followed twice a week by caliper measurements along two orthogonal axes: length (L) and width (W). The volume (V) of tumours was estimated by the following formula: V = L (W_2_)/2. Relative tumour volume (RTV) was calculated from the following formula: RTV = (Vx/V1), where Vx is the tumour volume on day X and V1 is the tumour volume at initiation of the treatment. Growth curve was obtained by plotting the mean volume of RTV on Y axis against time (X axis expressed as days after starting of treatment). Antitumour activity was evaluated according to tumour growth inhibition, calculated from the following formulae: percent GI = 100 − (RTVt/RTVc) × 100, where RTVt is the medium RTV of treated mice and RTVc is the median RTV of controls, both at a given time-point when the antitumour effect was optimal. At the time of killing, tumours were dissected out from the neighboring connective tissue, frozen and stored in nitrogen for further analysis.

### Histopathological analysis

Tumours, livers, lungs, spleens, and kidneys were fixed in 4% formalin, sectioned at 5 μm, and stained with hematoxylin and eosin Y, as suggested by the manufacturer (Bio-Optica, Milan, Italy).

### Immunohistochemical analysis

Paraffin embedded sections, 5 μm thick, were mounted on slides precoated with poly-lysine, and then they were deparaffinised and dehydrated (7–8 serial sections). Immunohistochemical experiments were performed with rabbit polyclonal Ki-67 primary antibody at 4°C overnight. Then, a biotinylated goat-anti-rabbit IgG was applied for 1 hr at room temperature, followed by the avidin biotin-horseradish peroxidase complex (ABC/HRP; Vector Laboratories, CA, USA). Immunoreactivity was visualized using the diaminobenzidine chromogen (DAB) (Sigma-Aldrich). Counterstaining was carried out with methylene-blue (Sigma-Aldrich). The primary antibody was replaced by normal rabbit serum in negative control sections.

### Statistical analysis

Data were analyzed for statistical significance (*P* < 0.05) using a two-tailed student's Test, performed by Graph Pad Prism 4. Standard deviations (SD) are shown.

## Results

### Design and synthesis of Peptide LDFI

Leptin, whose structure consists of 4 α helices (A-D helices), binds its receptor through three binding sites (I, ll and III) 45. The amino acid sequence 39-42, located in the loop that connects helices A and B, is essential for activation of the leptin receptor and represents the main target region that can be modified for obtaining ObR antagonists. Mutations of some or all of these amino acids to Ala in human and ovine leptin do not change their binding properties, but they abolish their biological activity and converts the muteins into potent antagonists 46,47. In this context and considering that the subsequence 39-42 represents a key residue in the activation of leptin-receptor complex, we designed and synthesized the small peptide derived from the wild-type sequence 39-42 of leptin Leu-Asp-Phe-Ile (LDFI). The tetrapeptide designed mimics the sequence of leptin binding site I, involved in the interaction with CRH2 (cytokine receptor homology domain) from the ObR and therefore in its activation. The tetrapeptide was synthesized by solid phase peptide synthesis, using Fmoc-chemistry. A ‘scramble’ tetrapeptide consisting of a random sequence of amino acids was also synthesized. Specifically the tetrapeptide Leu-Leu-Leu-Ala was prepared and its biological activity was tested by performing the same biological tests carried out for the tetrapeptide LDFI. The tetrapeptide was also synthesized in the pegylated form (LDFI-PEG). For this purpose the PEG was introduced through an amidic bond on the C-terminal amino acid, using a PEG with an amine linker.

### Peptide LDFI inhibits leptin-induced cell growth and motility in breast cancer cells

**Figure 1:**
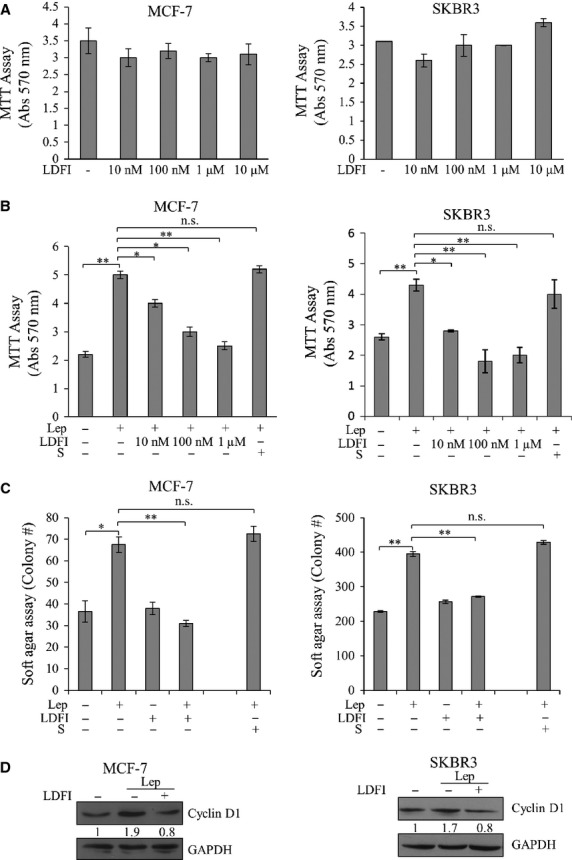
Effects of peptide LDFI on leptin-induced breast cancer cell proliferation. (A) MTT growth assays in MCF-7 and SKBR3 breast cancer cells treated with vehicle (-) or increasing doses of peptide LDFI (10 nM, 100 nM, 1 μM, 10 μM) for 96 hrs. (B) MTT growth assays in MCF-7 and SKBR3 cells treated with vehicle (-), leptin (Lep, 500 ng/ml), with or without peptide LDFI (10 nM, 100 nM, 1 μM) for 96 hrs. A scramble peptide (S, 1 μM) was used as negative control. (C) Soft agar anchorage-independent growth assays in MCF-7 and SKBR3 cells treated with vehicle (-), leptin (Lep, 500 ng/ml), alone or in combination with peptide LDFI (1 μM) or a scramble peptide (S, 1 μM). n.s. non-significant; *P < 0.05, **P < 0.01. (D) Immunoblotting for Cyclin D1 expression in MCF-7 and SKBR3 cells treated for 24 hrs with vehicle (-), leptin (500 ng/ml) with or without peptide LDFI (1 μM). GAPDH was used as control of equal loading and transfer. Numbers below the blots represent the average fold change in Cyclin D1/GAPDH ratio relative to vehicle-treated cells.

First, we tested the biological activity of the peptide LDFI on anchorage-dependent cell proliferation using as experimental models ObR-positive and leptin-sensitive MCF-7 (ERα-positive) and SKBR3 (ERα-negative) breast cancer cells. Both MCF-7 and SKBR3 cells were treated with the peptide at increasing concentrations (10 nM, 100 nM, 1 μM and 10 μM) for 96 hrs. The peptide did not interfere with cell proliferation in the absence of leptin and did not produce any significant cytotoxic effects at all the doses tested (Fig.1A).

Next, we explored the ability of peptide LDFI to interfere with leptin-induced cell proliferation (Fig.1B). As expected, in both cell lines treatment with leptin (500 ng/ml) increased cell proliferation. The peptide LDFI at 10 nM–1 μM concentrations significantly reversed the leptin-induced cell growth in a dose-dependent manner. The ability of peptide LDFI to inhibit leptin-mediated cell growth was also evaluated using anchorage-independent growth assays, which better reflect *in vivo* three-dimensional tumour growth (Fig.1C). The peptide significantly reduced the increase in colony numbers induced by leptin in MCF-7 and SKBR3 cell lines. In contrast, a scramble peptide (S), consisting of a random sequence of amino acids, used as a negative control, showed no leptin antagonistic properties. Data from growth assays were well correlated with a reduction in leptin-induced expression of Cyclin D1, a well known marker for cell proliferation, in cells treated with the peptide (Fig.1D).

**Figure 2:**
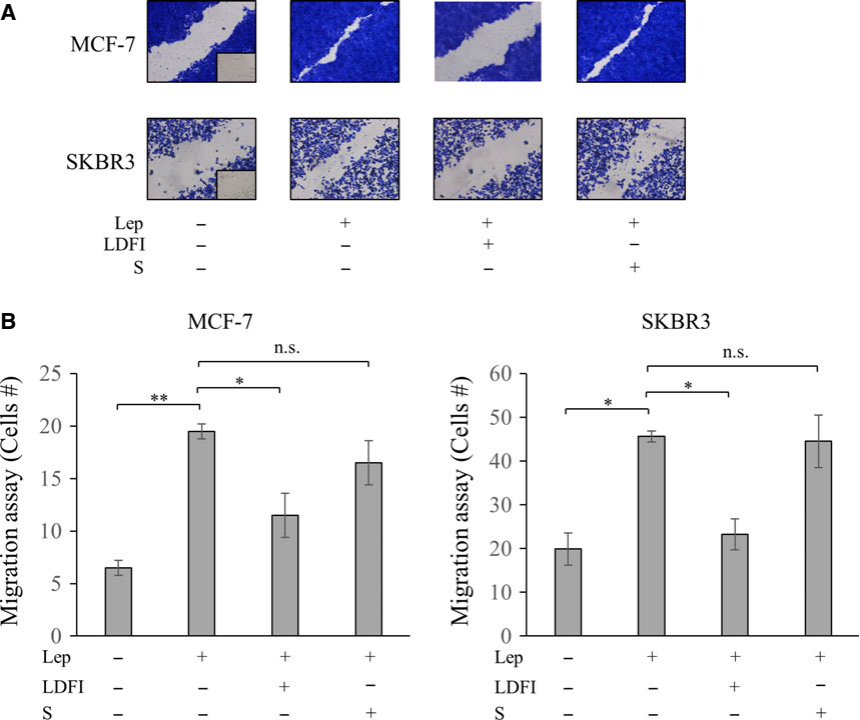
Peptide LDFI inhibits leptin-induced cell motility. (A) MCF-7 and SKBR3 breast cancer cells were subjected to wound-healing migration assays with images captured at 0 and 24 hrs after incubation with vehicle (-), leptin (Lep, 500 ng/ml) alone or in combination with peptide LDFI (1 μM) or a scramble peptide (S, 1 μM) using phase-contrast microscopy. Small squares, time 0. (B) Transmigration assays in MCF-7 and SKBR3 cells treated as in A. n.s. non-significant; *P < 0.05, **P < 0.005.

Moreover, we assessed, in MCF-7 and SKBR3 cells, the ability of the peptide to inhibit cell motility induced by leptin in wound-healing scratch assays. As shown in Figure2A, both cell lines moved the farthest in eitheir directions to close the ‘gap’ following leptin treatment compared to vehicle control conditions. Pretreatment with peptide LDFI counteracted leptin effects on cell motility. Then, the capacity of cells to migrate across uncoated membrane in transmigration assays was tested in the presence of leptin and peptide LDFI (Fig.2B). Leptin increased the number of migrated cells in both cell lines and again pretreatment with peptide LDFI resulted in a clear reduction in leptin-induced cell motility. In both wound-healing scratch and transmigration assays the exposure to the scramble peptide (S) did not influence leptin-induced effects on MCF-7 and SKBR3 cells (Fig.2A and B).

### Peptide LDFI blocks leptin signalling pathways

**Figure 3:**
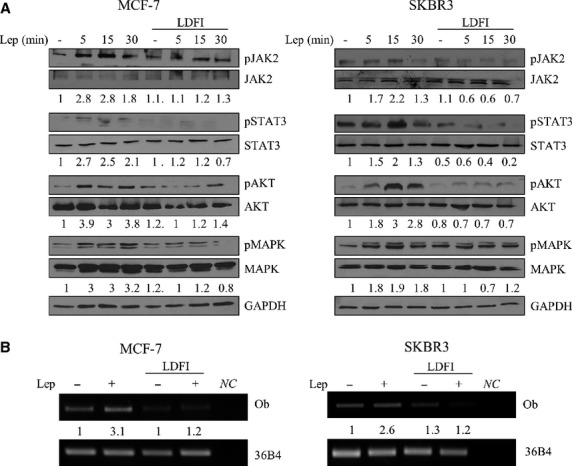
Peptide LDFI antagonizes leptin signalling activation. (A) Immunoblotting of phosphorylated (p) JAK2, STAT3, AKT, MAPK and total proteins from cells treated with vehicle (-), leptin (Lep, 500 ng/ml for 5, 15 and 30 min.) with or without peptide LDFI (1 μM). GAPDH was used as control of equal loading and transfer. Numbers below the blots represent the average fold change in phospho-proteins/total proteins/GAPDH ratio relative to vehicle-treated cells. (B) RT-PCR for leptin (Ob) and 36B4 (internal standard) mRNA expression in cells treated for 24 hrs with vehicle (-), leptin (Lep, 500 ng/ml) with or without peptide LDFI (1 μM). Numbers represent the average fold change in Ob/36B4 levels relative to vehicle-treated cells. NC, negative control.

Leptin exerts its biologic function through binding to its receptor which mediates a downstream signal by activating multiple signalling pathways. Thus, we conducted time-course studies to examine the effects of peptide LDFI on phosphorylation of the major leptin signalling molecules, that are known to mediate proliferation and motility in breast cancer cells, using immunoblot analysis (Fig.3A). As expected, in MCF-7 and SKBR3 cells, leptin treatment induced phosphorylation of JAK2/STAT3, AKT and MAPK, whereas pretreatment with peptide LDFI completely abrogated the leptin activation of these signalling pathways.

Since we have previously demonstrated that leptin was able to up-regulate its own gene expression 48, we investigated whether LDFI could also counteract this effect. RT-PCR analysis showed that the levels of the Ob gene increased by leptin were strongly reduced in the presence of the peptide in both breast cancer cells (Fig.3B). Moreover, we examined by real time PCR the expression of OBRL, OBRsh and VEGFR that are well-known leptin-induced genes. As expected, treatment with peptide LDFI was able to reverse the stimulatory effects induced by leptin on these genes (Fig. S1).

### Specifity of peptide LDFI in antagonizing leptin effects

**Figure 4:**
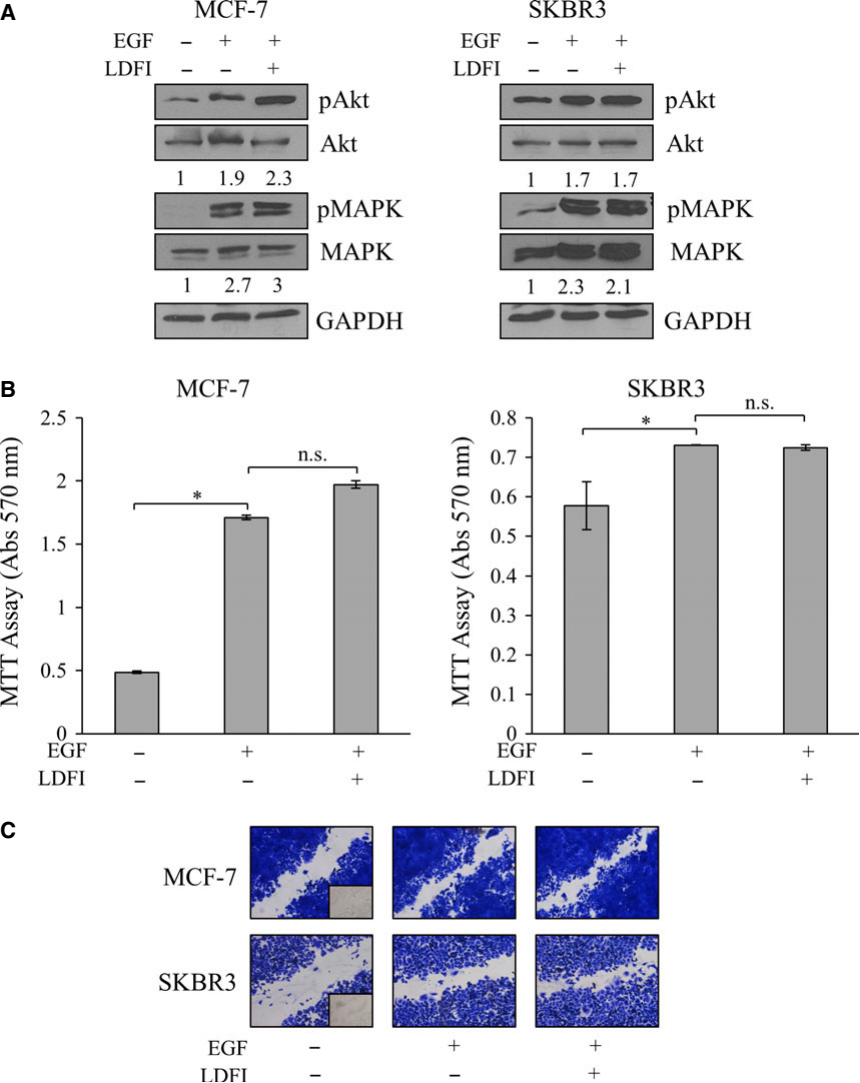
LDFI effects are specific for leptin signalling. (A) Immunoblotting of phosphorylated (p) AKT, MAPK and total proteins from cells treated with vehicle (-), EGF (100 ng/ml, for 5 min.) with or without peptide LDFI (1 μM). GAPDH was used as control of equal loading and transfer. Numbers below the blots represent the average fold change in phospho-proteins/total proteins/GAPDH ratio relative to vehicle-treated cells. (B) MTT growth assays in MCF-7 and SKBR3 cells treated with vehicle (-), EGF (100 ng/ml) alone or in combination with peptide LDFI (1 μM) for 96 hrs. ns, non-significant, *P < 0.05. (C) Cells were subjected to wound-healing migration assays with images captured at 0 and 24 hrs after incubation with vehicle (-), EGF (100 ng/ml) with or without peptide (LDFI, 1 μM) using phase-contrast microscopy. Small squares, time 0.

To test if LDFI action was specific for leptin effects, cell biological assays were performed in cells treated with a molecule able to elicit cellular responses through a different mechanism from leptin, such as EGF. Thus, we investigated, in MCF-7 and SKBR3 cell lines pretreated with LDFI, the effect of short-term stimulation with EGF (100 ng/ml) on phosphorylation levels of AKT and MAPK, the main downstream effectors of the growth factor signalling. The enhanced AKT and MAPK phoshorylation observed after treatment with EGF was not affected by the peptide (Fig.4A). In addition, data obtained from MTT growth assays (Fig.4B) and wound-healing scratch assays (Fig.4C) revealed that LDFI treatment did not reverse EGF-induced cell growth and motility.

### Efficacy of LDFI-PEG treatment in breast cancer xenograft models

As a final step of this study, we assessed the therapeutic potential of the novel ObR antagonist LDFI by evaluating the efficacy of the peptide in mouse xenograft models. To this aim, we developed a pegylated leptin receptor antagonist (LDFI-PEG) to increase the peptide bioavailability. Pegylation may result in improved *in vivo* potency related to a better stability, greater protection against proteolytic degradation and lower clearance. After verifying the structure and the purity of LDFI-PEG, we tested its effects on cell growth and motility in MCF-7 and SKBR3 cells (Fig. S2A and B). The LDFI-PEG peptide showed comparable biological activity with native LDFI in inhibiting leptin-induced cell proliferation and migration in both cell lines.

Therefore, we then used the SKBR3 orthotopic xenograft model to examine the effects of peptide LDFI-PEG on tumour growth *in vivo*. We injected SKBR3 breast cancer cells into the intrascapular region of female nude mice and followed tumour growth after administration of LDFI-PEG at 1 and 10 mg/kg/day. As SKBR3 cells can produce endogenous leptin, exogenous leptin was not inoculated into the mice. LDFI-PEG was not toxic and did not affect the energy balance since no change in body weight or in food and water consumption was observed. In addition, no significant differences in the mean weights or histological features of the major organs (liver, lung, spleen and kidney) after sacrifice were observed between vehicle-treated mice and those that received treatment.

**Figure 5:**
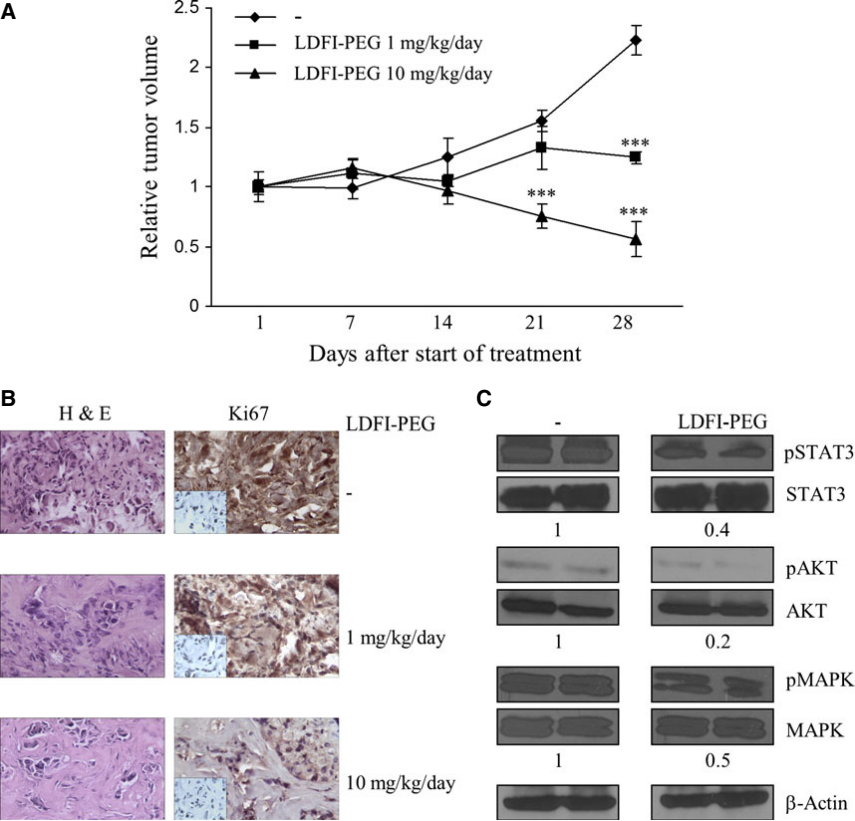
Impact of LDFI-PEG treatment on tumour growth of SKBR3 xenografts. (A) SKBR3 cells were inoculated into the intrascapular region of female nude mice (15 mice) and then treated 5 days a week with vehicle (-) or peptide PEG-LDFI (1 and 10 mg/kg/day) by intraperitoneal injection for 28 days (5 mice each group). Relative tumour volume (RTV) was calculated from the following formula: RTV=(Vx/V1), where Vx is the tumour volume on day X and V1 is the tumour volume at initation of the treatment (day 1). ***P < 0.0001. (B) Haematoxylin and eosin, Ki-67 staining of tumour sections from vehicle (-) and LDFI-PEG treated mice. Small squares, negative control. (C) Immunoblotting of phosphorylated (p) STAT3, AKT, MAPK and total proteins from tumours excised from vehicle (-) and LDFI-PEG treated mice (10mg/kg/day). β-actin was used as control of equal loading and transfer. Numbers below the blots represent the average fold change in phospho-proteins/total proteins/β-actin ratio relative to vehicle-treated mice.

Tumour volume was measured from the first day of treatment and the RTV was calculated as described in details in Materials and Methods. As shown in Figure5A, after LDFI-PEG treatment tumour volumes continued to reduce over control for the duration of experiment. Particularly, at the end of treatment (28 days) we observed that both dosages of PEG-LDFI induced a significant tumour growth inhibition (44% and 74.7% respectively) compared to vehicle-treated mice, although to a higher extent after treatment with 10 mg/kg/day. To determine whether the reduction in breast tumour growth induced by treatment with PEG-LDFI was associated with any changes in the mitotic index, we evaluated in tumours the expression of Ki-67 as a marker of proliferation. Sections of tumours from PEG-LDFI-treated mice exhibited a dose-dependent reduction in the expression of Ki-67 compared with that in tumours from vehicle-treated mice (Fig.5B). In addition, immunoblot analysis revealed that the phosphorylation levels of STAT3, MAPK and AKT were significantly lower in SKBR3 xenograft tumours from mice treated with LDFI-PEG than in tumours from vehicle-treated controls (Fig.5C).

## Discussion

The critical role played by leptin in mammary tumourigenesis has generated a great interest in the design and development of several leptin signalling modulators that could interfere with the action of leptin and thereby prevent or delay breast cancer development and progression. The most important issue in modulating leptin pathways is to achieve target specificity since this adipokine does not only influence cancer tissues, but it is implicated in a wide spectrum of physiological processes in peripheral organs as well as in the central nervous system.

Biological actions of leptin are mediated through binding to the extracellular domain of specific leptin receptor (ObR) present in a variety of tissues and localized to the cell membranes. It has been reported that the structure of leptin resembles four alpha-helix bundle cytokines and ObR is a member of the class I cytokine receptor family. The ObR, encoded by *db* gene, includes six isoform (ObR_a-f_), resulting from alternative splicing: the long isoform with full intracellular signalling capabilities and shorter isoforms with less biological activities. The large extracellular domain of ObR (816 amino acids) is common to all ObR isoforms, and the variable length cytoplasmic tail (300 amino acids residues) distinguished the several isoforms 21,49. Leptin interacts with its receptor through three potential binding sites. Binding site I is located around leptin residue 40, the bivalent site II consists of a leptin domain around the N-terminus (aa 3–21) and another in the middle (aa 70–93), and site III is positioned at the leptin's C terminus (aa 110–142) 45. Even though the three-dimensional structure of leptin was elucidated a few years after its discovery 50, the crystallographic structure of leptin-leptin receptor complex has not been reported yet. Until today, all data available are based on theoretical models 45,51 from which it is clear that the amino acid sequence 39-42, located in the loop that connects helices A and B, constitutes a key sequence in the leptin-receptor-binding site. Several groups have developed short leptin fragments containing specific ObR interacting domains that demonstrated anti-neoplastic activity both *in vitro* and *in vivo* cancer models 31–35.

Here, we generated a four amino acids peptide corresponding to leptin wild-type sequence 39-42 that plays a crucial role in the activation of ObR. Our results showed that the novel ObR antagonist peptide LDFI inhibits the leptin-induced anchorage-dependent and -independent growth as well as migration in both ERα-positive and -negative breast cancer cells without exhibiting any partial agonistic activity in the absence of leptin. The anti-tumour action of LDFI was associated with the inhibition of several leptin-induced pathways such as JAK2, STAT3, AKT and MAPK and a reduction in Cyclin D1 expression. Interestingly, we demonstrated the ability of the peptide LDFI to reverse the leptin-mediated up-regulation of its own gene expression underlying how this peptide negatively interferes in the short autocrine loop maintained by leptin on Ob gene in breast cancer cells. The described effects were specific for leptin signalling since the developed peptide was not able to antagonize the other growth factor's actions on signalling activation, proliferation and migration.

To assess the clinical utility of our leptin antagonist against human breast cancer progression, we tested LDFI effects in SKBR3 xenografts implanted in female nude mice. Because the peptide normally would have a relatively short biological half-life, we pegylated it to increase its bioavailability and potentiate its effects. Indeed, covalent modification with high molecular weight PEG chains is a very efficient method for improving the pharmacokinetics of biomolecules 52 and has been shown to increase the half-life of wild-type leptin 53–55. Several PEG-conjugated medications have proven to be superior to their unmodified parent molecules and they are now widely used in clinical practice 56,57. A significant growth reduction in SKBR3 xenografts was found after LDFI-PEG treatment. Moreover, we observed in tumour sections from LDFI-treated mice a marked decrease in the expression of the nuclear proliferation antigen Ki-67 as well as in the phosphorylation levels of leptin downstream effectors. Importantly, in mice, LDFI produced no signs of systemic toxicity and did not affect the energy balance. Indeed, no significant effects on body weight were found between vehicle-treated and LDFI-PEG-treated mice.

Overall, our data demonstrate that LDFI may represent a novel leptin receptor antagonist able to reduce breast cancer progression both *in vitro* and *in vivo*, suggesting its potential use in the treatment of breast cancer, especially in obese women.

## Supporting Information

**Figure S1** LDFI effects on leptin-induced genes.

**Figure S2** LDFI-PEG effects on breast cancer cell growth and motility.

**Data S1** Table of content of NMR (S2-S5) and RP U-HPLC/MS (S6-S8) analyses of the peptides used in the present study.
