# Supplementary figures and images for: A novel leptin antagonist peptide inhibits breast cancer growth in vitro and in vivo
Source: J Cell Mol Med. 2015 Feb 27;19(5):1122–32. doi: 10.1111/jcmm.12517 (PMC4420614; doi:10.1111/jcmm.12517)

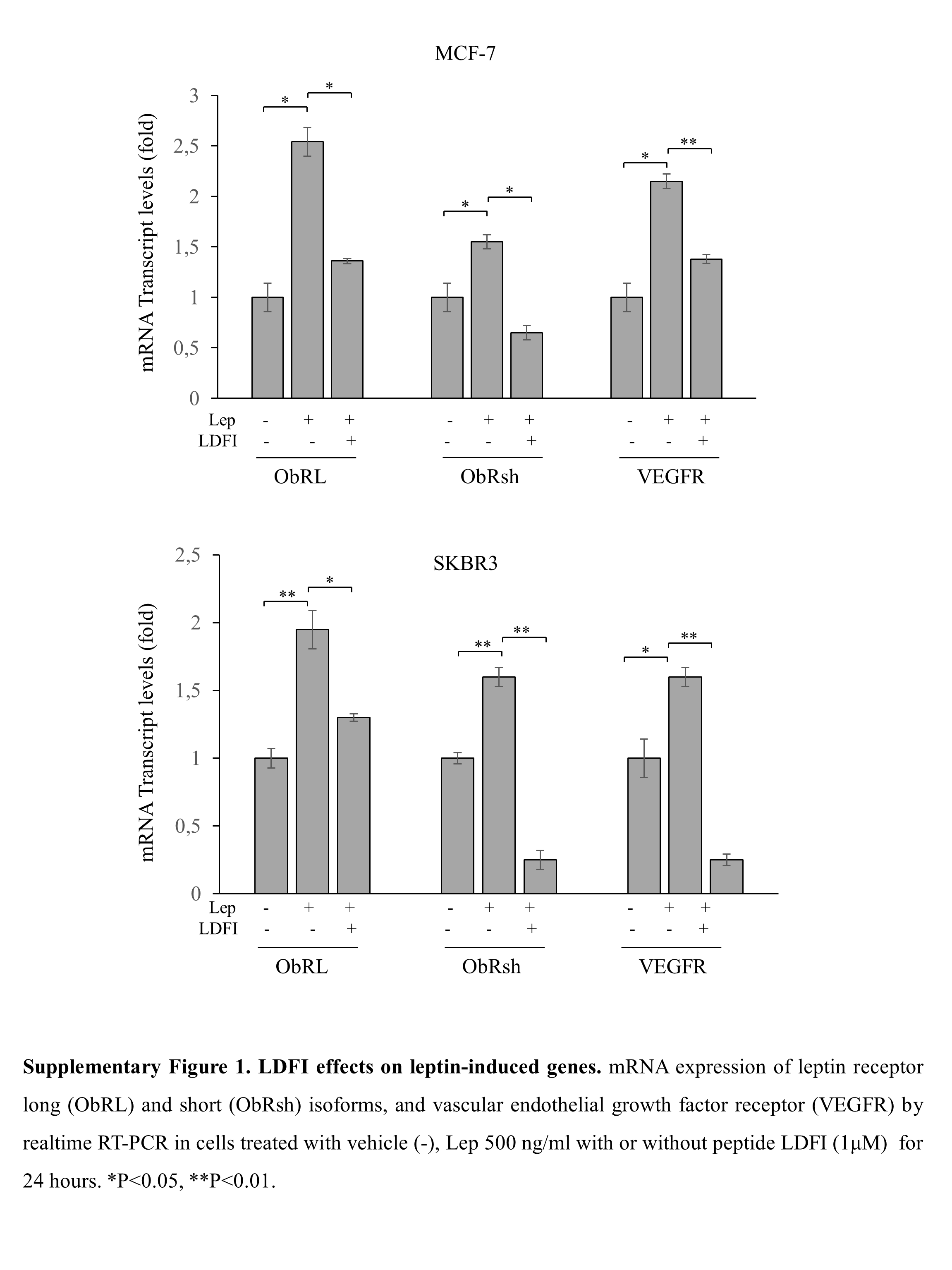

Supplement: Supplementary file 1 [file jcmm0019-1122-sd1.tif]

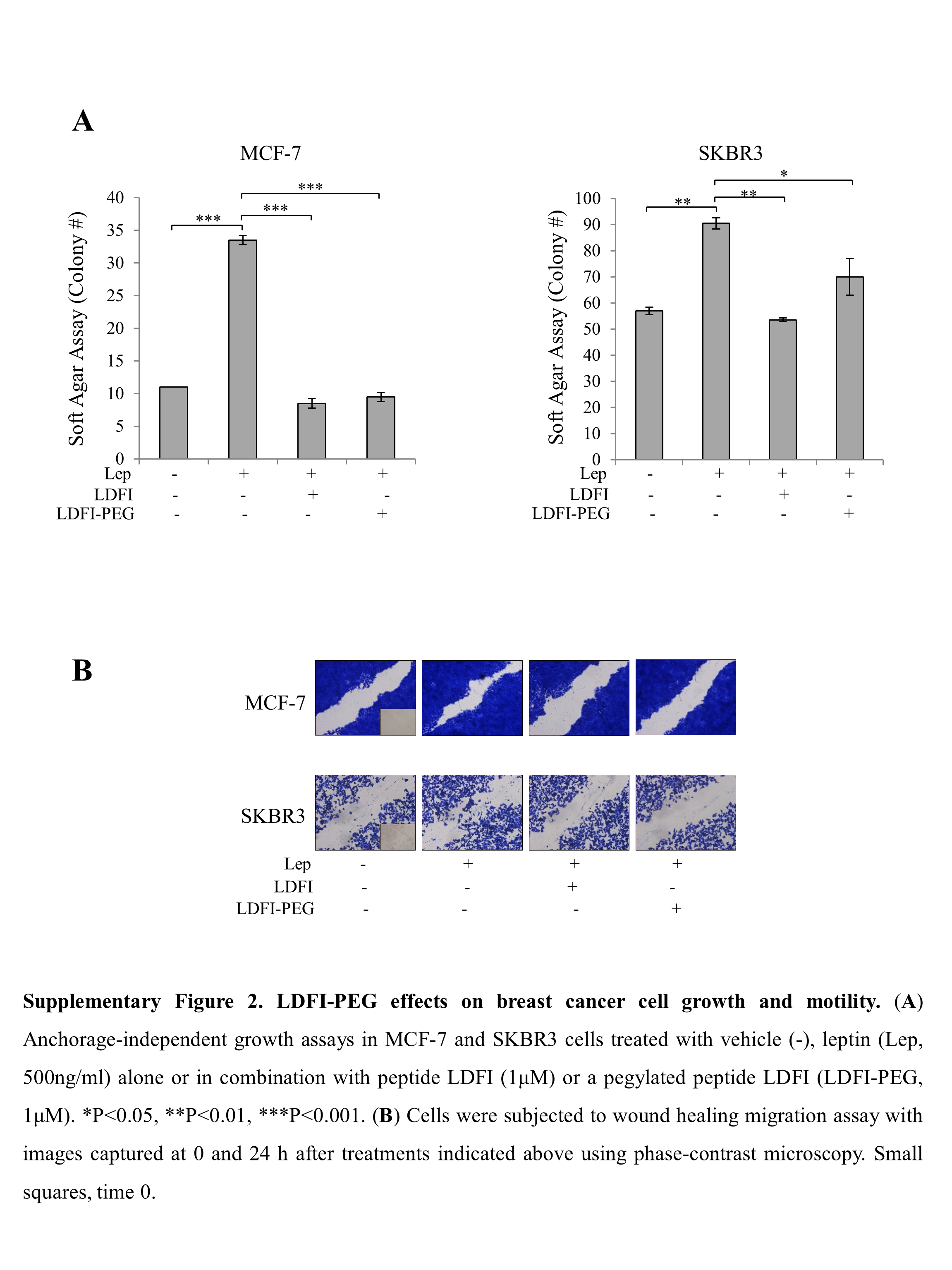

Supplement: Supplementary file 2 [file jcmm0019-1122-sd2.tif]
