# Supplementary material for: A novel leptin antagonist peptide inhibits breast cancer growth in vitro and in vivo
Source: J Cell Mol Med. 2015 Feb 27;19(5):1122–32. doi: 10.1111/jcmm.12517 (PMC4420614; doi:10.1111/jcmm.12517)
Supplement: Supplementary file 3 [file jcmm0019-1122-sd3.doc]

**Supporting Information**

**A novel leptin antagonist peptide inhibits breast cancer growth *in vitro* and *in vivo***

Stefania Catalano^a,#^, Antonella Leggio^a,#^, Ines Barone^a^, Rosaria De Marco^a^, Luca Gelsomino^b^, Antonella Campana^a^, Rocco Malivindi^a^, Salvatore Panza^a^, Cinzia Giordano^c^, Alessia Liguori^d^, Daniela Bonofiglio^a^, Angelo Liguori ^a,*^, Sebastiano Andò^a,c,*^

^a^ Department of Pharmacy, Health and Nutritional Sciences, University of Calabria, Arcavacata di Rende, CS, Italy

^b^ Breast Center, Baylor College of Medicine, Houston, Texas, USA

^c^ Centro Sanitario, University of Calabria, Arcavacata di Rende, CS, Italy

^d^ Department of Medical Oncology, University of Messina, Italy

TABLE OF CONTENTS

^1^H NMR spectrum for **LDFI** peptide **S2**

^13^C NMR spectrum for **LDFI** peptide **S3**

^1^H NMR spectrum for “scramble” peptide **LLLA S4**

^13^C NMR spectrum for “scramble” peptide **LLLA S5**

RP U-HPLC/MS analysis and ESI-QTOF-MS/MS spectrum for **LDFI** peptide **S6**

RP U-HPLC/MS analysis for “scramble” peptide **LLLA S7**

ESI-QTOF-MS/MS spectrum for **LDFI-PEG** peptide **S8**

S1

**^1^H NMR (300 MHz, DMSO-*d_6_*) spectrum of LDFI peptide**

**^1^H NMR (300 MHz, DMSO-*d_6_*):** δ 8.60 (s_broad_, NH, 1H), 8.07 (d, J = 8.1 Hz, NH, 1H), 7.99 (d, J = 8.5 Hz, NH, 1H), 7.29-7.11 (m, Ar-H, 5H), 4.62-4.50 (m, α-CH_Phe_ + α-CH_Asp_, 2H), 4.18 (dd, J = 6.0 Hz, J = 8.5 Hz, α-CH_Ile_, 1H), 3.66 (m, α-CH_Leu_, 1H), 3.06 (dd, J = 4.0 Hz, J = 14.3 Hz, C*H*_2_Phe, 1H), 2.82 (dd, J = 9.6 Hz, J = 14.3 Hz, C*H*_2_Phe, 1H), 2.66 (dd, J = 5.6 Hz, J = 16.6 Hz, C*H_2_*COOH, 1H), 2.51-2.42 (m, C*H*_2_COOH, 1H), 1.79 (m, β-CH_Ile_, 1H), 1.62 (m, γ-CH_Leu_, 1H), 1.51-1.39 (m, CH_2Leu_ + CH_2Ile_, 3H), 1.19 (m, CH_2Ile_, 1H), 0.95-0.81 (m, CH_3Ile_ + CH_3Leu_, 12H)

S2

**^13^C NMR (75 MHz, DMSO-*d_6_*) spectrum of LDFI peptide**

**^13^C NMR (75 MHz, DMSO-*d_6_*):** δ 173.16, 171.89, 171.15, 170.43, 169.55, 138.01, 129.60, 128.42, 126.66, 56.93, 54.08, 51.15, 50.08, 37.67, 36.87, 25.14, 23.81, 23.30, 21.91, 18.28, 15.95, 11.72

S3

**^1^H NMR (300 MHz, DMSO- *d_6_*) spectrum of Scramble peptide LLLA**

**^1^H NMR (300 MHz, DMSO-*d_6_*):** δ 8.58 (d, J = 8.4 Hz, NH, 1H), 8.18-8.08 (m, NH, NH_2_, 4H), 4.48-4.37 (m, α-CH_Leu_, 1H), 4.37-4.28 (m, α-CH_Leu_, 1H), 4.15 (m, α-CH_Ala_, 1H), 3.78 (m, α-CH_Leu_, 1H), 1.68-1.54 (m, γCH_Leu_, 3H), 1.52-1.39 (m, βCH_2Leu_, 6H), 1.25 (d, J = 7.2 Hz, CH_3Ala_, 3H), 0.93-0.79(m, CH_3Leu_, 18H)

S4

**^13^C NMR (75 MHz, DMSO-*d_6_*) spectrum of Scramble peptide LLLA**

**^13^C NMR (75 MHz, DMSO-*d_6_*):** δ 174.34, 171.98, 171.49, 169.00, 51.44, 51.14, 51.00, 47.84, 24.46, 24.40, 23.89, 23.60, 23.42, 22.99, 22.52, 22.26, 21.79, 17.54.

S5

**RP U-HPLC/MS analysis of LDFI peptide**

**
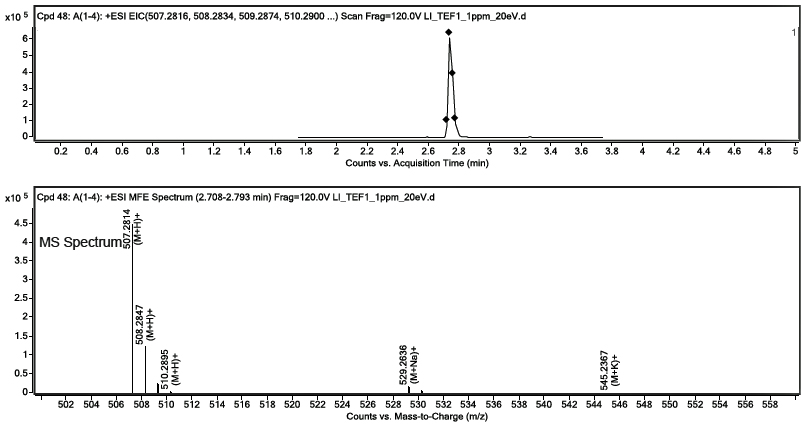
**

**ESI-QTOF-MS/MS spectrum for LDFI peptide**


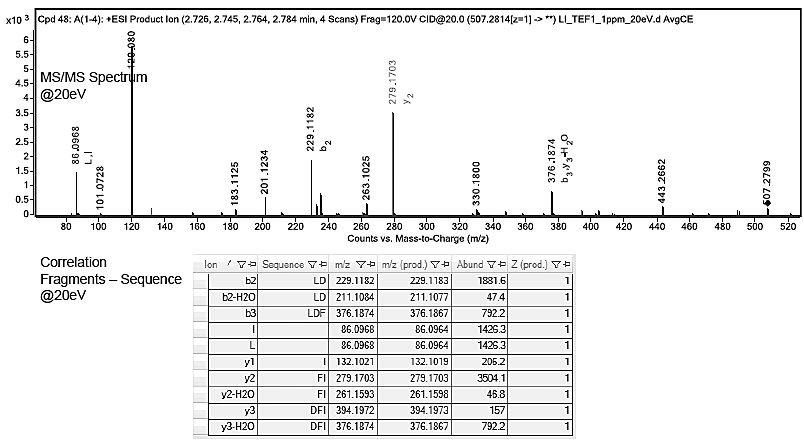


S6

**RP U-HPLC/MS analysis of scrambled peptide LLLA**

**
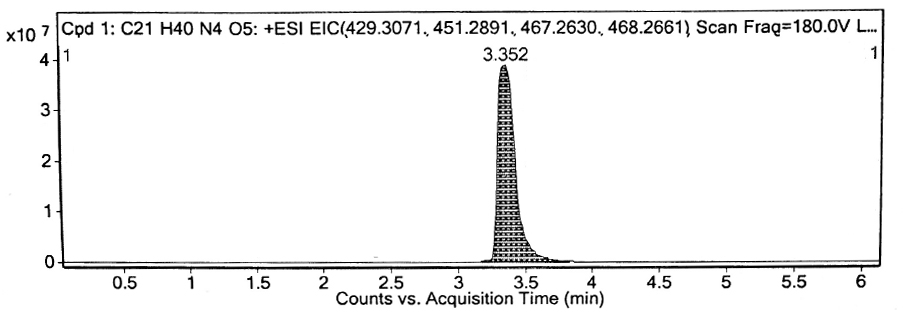
**

**
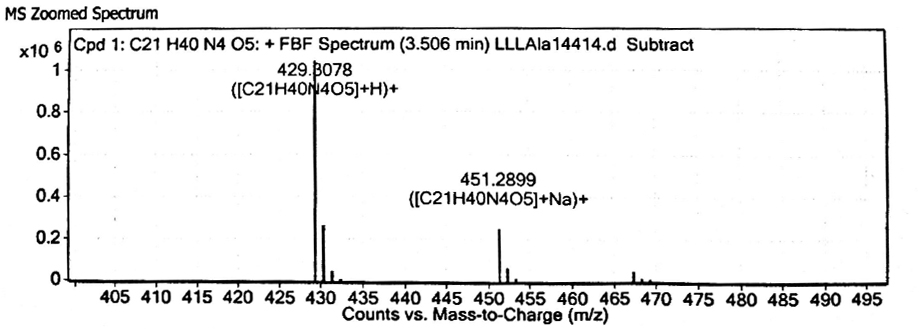
**

S7

**ESI-QTOF-MS/MS spectrum for LDFI-PEG peptide**

**
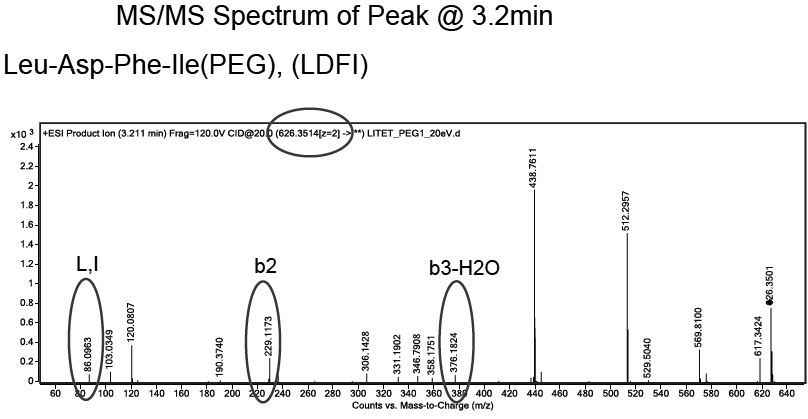
**

S8
